# Supplementary material for: Well-Being Workshops in Eating Disorder Wards and Their Perceived Benefits to Patients and the Multi-Disciplinary Team: A Pilot Study
Source: Brain Sci. 2019 Sep 23;9(10):247. doi: 10.3390/brainsci9100247 (PMC6826568; doi:10.3390/brainsci9100247)
Supplement: Supplementary file 1 [file brainsci-09-00247-s001.pdf]

Supplementary Material 1: Well-being workshop questionnaire

Please indicate the extent to which you agree with each statement by circling the number from 1 to 5.
1 = completely disagree
5 = agree completely

I enjoy taking time for myself

1	2	3	4	5

Taking time out for myself feels good

1	2	3	4	5

I feel that I deserve to take time out for myself

1	2	3	4	5

I don’t take time out for myself very often

1	2	3	4	5

Today I am in a good mood

1	2	3	4	5

Supplementary Material 2: Feedback Questionnaire

Initials……………………

Group Feedback Questionnaire – Well-being Workshop

1.) How much did you enjoy this event?

Did not enjoy at all		quite enjoyed it		really enjoyed
**1**	**2**	**3**	**4**	**5**

2.) How useful was this workshop?

Not useful at all		quite useful		really useful
**1**	**2**	**3**	**4**	**5**

3.) How relevant was the workshop content to your own self-care practices?

Not at all relevant		some relevance		very relevant
**1**	**2**	**3**	**4**	**5**

4.) What did you like the most about the workshop?

……

……

……

5.) Please give your ideas about how we could improve the workshop in the future:

……

……

……

6.) Any other comments?

……

……

……

Supplementary Material 3: Open-ended questions

Questions for Patient focus group

1. What have you thought of the wellbeing workshops?
2. What do you think about the patient/ staff collaboration?
3. Do you think these workshops are important?
4. Do you think these wellbeing workshops have the potential to impact your recovery?
5. How do you think we could improve them to help your recovery?
6. How do you think these workshops impacted your wellbeing?
7. What do you personally do for your wellbeing?
8. Would you like to add anything further?

Questions for MDT Individual Interviews

1. What was your experience of the wellbeing workshops?
2. What do you think about the patient/ staff collaboration?
3. Why do you think these workshops are important?
4. Do you think these wellbeing workshops have the potential to impact patient recovery?
5. How do you think we could improve them to help patient recovery?
6. Do you have any ideas for future workshops we could run?
7. How do you think these workshops impacted your wellbeing?
8. What do you personally do for your wellbeing?
9. Tell us about job burn-out and how we can address it better?
10. Would you like to add anything further?

Supplementary Material 4: An extended table of qualitative examples for each theme for the MDT and patient groups

Theme	Patient examples	MDT Examples
Enjoyment	"I found it a lot of fun and a great positive distraction"<br><br>"I thought it was quite lacking"	"I actually really enjoyed the workshops. Taking that time out and doing something and coming back to work, it is so much more productive"<br><br>"I felt happier as well, and more sort of determined to get sort of work done"<br><br>"It has been a lovely oasis in the middle of quite a stressful day, for me."
Recovery & Well-being	"There is a huge need for them"<br><br>"I think they are a real opportunity"<br><br>"If its only one little thing that will make that difference between things going right and things not, it's just trying to find stuff that, and its normally external stuff like the well-being stuff that can be that good trigger, that's why it's really important."<br><br>"it's going beyond the illness then its almost like we are more than the illness"<br><br>"We are kind of reinstating our true identify away from this illness and I think that… in every workshop, in any situation, you do at the end of it take something back and reflect upon it and that impacts in the longer term beyond anorexia"	"They sort of teach that it's important to look after yourself and all the different aspects of yourself so I think in that sense they could help with recovery."<br><br>"taking care of yourself is fundamental to all human beings"<br><br>"I really feel that burn-out is directly correlated with the ability for staff to take time out or for staff to look after themselves in their well-being"<br><br>"It's interesting to notice that staff members and also sometimes the patients struggle to prioritise their well-being and take time out"<br><br>"In the NHS there is increasing demands all the time and I think one way can really help with that is well-being workshops as well, having support"<br><br>"I think it sends a huge message to the patients that even on a super busy day staff make time for their own well-being and manage the stresses and strains because well-being is a bit of a priority. I think modelling that is good for the patient."

		"I think for different reasons the patients and the staff carry a huge amount of stress and they do very serious things together a lot of the time and I think it makes it quite hard for them and something de-humanising about that process and the trap that staff fall into is seeing everyone as anorexia rather than the individual"
Relationships	"I guess if they are interacting with people, that's the big one, and it's hard because its sort of instinctive, you want to isolate a lot of the time"<br><br>"I think the more we can do to smash down that boundary."<br><br>"I think if there could be the idea that we are all there as equals so taking away the patient staff boundary and everyone is just there as an individual."<br><br>"it felt, still quite divided, like there's a division and I think that was because there was no common ground per se, it's just the facilitator engaging individuals whereas I think it may be a better way to run it for the facilitator to create group activities."	"The relationships and dynamics can be quite difficult between the staff and the patients….it builds up therapeutic relationship quicker"<br><br>"When they've worked best is when they've had a lot of engagement from patients and staff and it has really felt like they are doing something together"
Content	"I found it interesting and helpful to my situation"<br><br>"Learning something new"<br><br>"Focusing on something pleasurable for no reason other than its something that makes you happy"<br><br>"It seemed quite image-focused and I think that's something to try and steer away from in terms of activities."	"they got us to do were kind of like mini bonding exercises and there is something really magical about that because it was about a simple exercise like standing on one leg on your own and realising you are really wobbly and then you stand on one leg and everyone supports each other with a single hand and that is all you need and suddenly you realise we can all stand really steady in a circle when we support each other, so a really non verbal way of bonding and everybody talks about that afterwards… I think when the well-being workshops can bring about

	"Didn't know it was going to be as interactive or focus on the body- some warning would have been useful"<br><br>"I can see how all of these techniques are really positive and helpful, but I didn't feel comfortable/ ready for some of them. Would be good to revisit at a later date."	moments like that they work really well"<br><br>"It feels too lecturey. It's too set and too structured, and I often find the irony is that we talk about people being flexible and perfectionist, and I find that this place is too inflexible and too perfectionist"<br><br>"I think these workshops should focus on 'doing' for the process/ enjoyment/ trying something novel"
Scheduling	"You could have your little things going on all the time and it gets everyone engaged and feeling secure, it gets the staff and patients used to doing stuff together and then every month you have something a bit more like different where you face kind of more of an event or something,"<br><br>"If you don't get people warmed up nothing happens because everyone was too scared to come and it's very difficult, especially because your big event will probably be somewhere outside of the ward where everyone will already feel uncomfortable to start with."	"The only thing is finding time to get to them, that can be hard"<br><br>"Having more. I think having more, having them frequently as well"
Future Ideas	"Something to do more with sort of the basics of looking after yourself properly and well…you know just like little things"<br><br>"I would rather have more sort of activity led ones, sort of engaging ones…something that can inspire and you can take outside as well."<br><br>"I think from my perspective I find things useful sometimes when it comes from someone whose been through similar"	"Activities, like fun games because I really do think that people with EDs don't play"<br><br>"If you can do something like creative and just fun and getting like messy and maybe even making something"<br><br>"Pre-explaining/ planning the group in advance and being flexible to accommodation attendance should be encouraged"
